# Evaluating the Effect of Stimuli Color and Frequency on SSVEP

**DOI:** 10.3390/s21010117

**Published:** 2020-12-27

**Authors:** Xavier Duart, Eduardo Quiles, Ferran Suay, Nayibe Chio, Emilio García, Francisco Morant

**Affiliations:** 1 Departament de Psicobiologia, Facultat de Psicologia, Universitat de València, 46010 València, Spain; xavier.duart@uv.es (X.D.); ferran.suay@uv.es (F.S.); 2 Instituto de Automática e Informática Industrial, Universitat Politècnica de València, 46022 València, Spain; nachch@posgrado.upv.es (N.C.); egarciam@isa.upv.es (E.G.); fmorant@isa.upv.es (F.M.); 3 Facultad de Ingeniería, Ingeniería Mecatrónica, Universidad Autónoma de Bucaramanga, 680003 Bucaramanga, Colombia

**Keywords:** brain–computer interface, electroencephalography, biomedical signal processing, SSVEP

## Abstract

Brain–computer interfaces (BCI) can extract information about the subject’s intentions by registering and processing electroencephalographic (EEG) signals to generate actions on physical systems. Steady-state visual-evoked potentials (SSVEP) are produced when the subject stares at flashing visual stimuli. By means of spectral analysis and by measuring the signal-to-noise ratio (SNR) of its harmonic contents, the observed stimulus can be identified. Stimulus color matters, and some authors have proposed red because of its ability to capture attention, while others refuse it because it might induce epileptic seizures. Green has also been proposed and it is claimed that white may generate the best signals. Regarding frequency, middle frequencies are claimed to produce the best SNR, although high frequencies have not been thoroughly studied, and might be advantageous due to the lower spontaneous cerebral activity in this frequency band. Here, we show white, red, and green stimuli, at three frequencies: 5 (low), 12 (middle), and 30 (high) Hz to 42 subjects, and compare them in order to find which one can produce the best SNR. We aim to know if the response to white is as strong as the one to red, and also if the response to high frequency is as strong as the one triggered by lower frequencies. Attention has been measured with the Conner’s Continuous Performance Task version 2 (CPT-II) task, in order to search for a potential relationship between attentional capacity and the SNR previously obtained. An analysis of variance (ANOVA) shows the best SNR with the middle frequency, followed by the low, and finally the high one. White gives as good an SNR as red at 12 Hz and so does green at 5 Hz, with no differences at 30 Hz. These results suggest that middle frequencies are preferable and that using the red color can be avoided. Correlation analysis also show a correlation between attention and the SNR at low frequency, so suggesting that for the low frequencies, more attentional capacity leads to better results.

## 1. Introduction

A brain–computer interface (BCI) is a system that allows a direct communication between the brain and the external world since it directly translates recorded neural activity into a control signal for an external device [1,2,3,4,5,6,7]. It can also be defined as a system that provides an interface to communicate or control a physical environment, through the use of brain signals, and without using the normal neuro-muscular pathways [2,6,8]. In short, it is a system that allows interaction between people and physical systems, such as computers, wheelchairs or other devices, through forms of interaction that do not involve the musculoskeletal system [9,10]. The most important application of BCIs in their few decades of existence has been to allow subjects with physical disabilities to interact with external devices [11,12,13,14]. A person could be confined to bed and from there turn on and off lights, heating, television, home automation, speller or any other device by BCI control [15,16,17,18]. Therefore, it is an applied field where neuroscience and technology converge, and it is of paramount importance if the aim is to improve the quality of life of people with this type of need [8].

BCIs extract information from electroencephalographic signals measured by means of applying mostly non-invasive electrodes to the participant’s scalp [12]. Various paradigms are used to obtain information about the wishes and intentions of the participant, which is necessary to generate useful commands oriented to fulfill the BCI purposes [13]. In the steady-state visual-evoked potentials (SSVEP) paradigm employed in this study, participants are presented with visual stimuli in basic shapes, such as circles or squares, in computer screens or dedicated LEDs [1]. These stimuli flicker at different frequencies, and observing them triggers evoked potentials, resulting from the electrical activity of neurons in the visual cortex [19,20]. This response occurs at the same frequency as the blinking stimulus, and also, with a lower intensity, at the frequencies of its harmonics which are multiples of the fundamental one [21]. A typical arrangement may include several stimuli flashing at different frequencies, so that each one represents a different commandment that the participants may choose by means of directing their gaze [19,20]. By measuring and recording the evoked activity by means of electroencephalography (EEG) devices and applying signal processing and spectral analysis techniques—frequency domain analysis—it is possible to determine with relative ease which stimulus is the participant observing, as well as selecting the control action which corresponds to their desires [19]. For a reliable operation of the system, however, a correct identification of the stimulus that the subject is looking at is needed, as well as reducing as much as possible the possibility to obtain false positives. This will allow us to undertake the right action on the devices, depending on the participant’s wishes.

The process of detecting and discriminating frequency components in SSVEPs is influenced by both their amplitude and the noise accompanying them. It should be borne in mind that noise is everything that does not correspond to the signal we aim to detect, and can be formed by both external disturbances that interfere with the process of capturing signals, and internal disturbances due to the incessant activity produced by the brain, as well as by artifacts due to blinking or to participant’s movements [20]. The relationship between the signal level and the noise level (signal-to-noise ratio, SNR) is a measure that allows us to know precisely how large the amplitude of the evoked signal is in relation to the noise level or background activity [22]. Higher SNR values help to better detect and discriminate the frequency components of interest to us [20,23,24,25].

Several stimulus properties can influence the amplitude of the harmonics in the obtained SSVEPs, as well as the capacity to discriminate them from the noise to which they are superimposed. Among theses properties there is the color of the stimulus and the blinking frequency. They can also affect the comfort experienced by the user; a factor that may affect the results obtained. Prolonged observation of blinking stimuli causes visual fatigue [19,26,27,28], which provokes decreases in the amplitude of visual-evoked potentials. It has been reported that certain colors, such as red, and certain low frequencies, can induce epileptic seizures in some people [20,29]. Some studies have addressed the comparison of these properties in order to draw conclusions applicable to the design of reliable BCIs. For the time being, however, there is no consensus on which are the best, and a high interindividual variability has been reported in the responses to these stimuli [19].

Some researchers [26] have investigated the influence on SSVEP of five-colors—white, gray, red, green and blue—at different frequencies between 7.5 and 17.14 Hz. To that end, they have studied the amplitude of the evoked frequencies and also the harmonics’ phase, by means of the canonical correlation analysis (ACC). The results show that white generates both the largest amplitude and the smallest phase variance; two favorable conditions to increase the discriminability of the stimuli, and to facilitate a higher performance of the final system. Gray follows white, then red and green, and finally blue. In contrast, in [29] the authors conclude that red is the color that provides the highest discriminating accuracy, although they point out as drawbacks that it is the least comfortable, and that according to the literature it can be dangerous because of the possibility of inducing epileptic seizures [30,31]. Finally, and for security reasons, they suggest the green color as the most appropriate to design BCI systems with a SSVEP paradigm. On the other hand, in [21] researchers tested 10 colors by means of spectral analysis for amplitudes, concluding that colors with large wavelengths, such as red and orange, would capture more attention and generate SSVEP of a higher amplitude, than shorter wavelength colors such as blue and purple. In a review of 57 articles on visual stimulation in BCI systems with the SSVEP paradigm, in [19] researchers classified stimulation frequencies into three bands: low (1–12 Hz), medium (12–30 Hz), and high (30–60 Hz). The low and medium frequencies have, according to the authors, the disadvantage of causing more visual fatigue, and of interfering with the spontaneous activity of the brain, while the high ones are better in these aspects, although they generate SSVEP of a lesser amplitude. The authors note that these high frequencies have received little consideration and should be further investigated. In terms of the colors, they indicate that red causes high-amplitude SSVEP at 11 Hz, but this amplitude decays with both lower and higher frequencies. Blue, on the other hand, shows a flatter response with frequency, but also less amplitude. According to the authors, there are no clear conclusions about which color is preferable, and perhaps the color selection should be chosen in order suit the participants’ characteristics.

Another potentially important factor in order to obtain evoked visual potential is attention. The sensory environment includes a large number of stimuli, and each one is a potential focus for attention. Only a few, however, are behaviorally relevant at any given time. Attention is in part a mechanism aimed to select the most relevant environmental characteristics for current or planned behavior [32]. In relation to visual perception, attention acts as a modulating factor on the neuronal activity of the visual cortex. Attention to visual stimuli increases cortical neurons’ responses, and attention directed to a particular site in the visual field improves detection and discrimination, and also reduces reaction times in that position relative to others [33]. Not only is the response to the attended stimuli, in the visual cortex, increased but also the suppressive influences of nearby distractors are decreased [34]. Concentration in the fovea field enhances the evoked visual potentials generated by stimulation in that field, while reducing the potentials generated in the peripheral visual field [35]. Attention thus influences the amplitude of the evoked visual potentials, participants with low attentional capacity might show poorer performances while using such devices (i.e., generating SSVEP with worse amplitude and reducing the percentage of correct positives in classifying tasks). In [36], the context of BCIs with motor imagery paradigm (another paradigm that requires great imaginative abilities), suggest that some previous training might improve attentional ability and consequently performance with BCI devices. As has been shown, although these are different paradigms, attention is also a very important factor here, and therefore it is advisable to study the relationship between attentional capacity and amplitude of SSVEP, when considering training oriented to an effective use of these devices.

The main objective of this study is to compare the properties of visual stimuli of different colors and frequencies, in terms of their discriminability, measured as a SNR, in an offline task (i.e., the subject observes the stimuli and EEG signals are recorded for later analysis, but no classification decisions are made, nor action taken on devices, as would be the case with an online task) corresponding to the SSVEP paradigm. Three of the most widely employed and recommended colors in the literature are used: red, green, and white, and three frequencies corresponding to the low, medium, and high bands according to the classification of [19,37]: 5, 12 and 30 Hz. As specific objectives, we aim to check whether the SNR obtained with the white color is comparable to that obtained with the red, because, for safety reasons, it may be preferable not to use the latter. On the other hand, the comparison of the different frequencies will allow us to check the possible advantage of working with high frequencies; a range of frequencies in which brain activity interferes less with frequency recognition. We also aim to verify the existence of relationships between SSVEP activity and several variables related to attentional capacity. We propose as a hypothesis that the white color can reach SNR of the same magnitude as the red, which would help to dispense with this color in real-life applications. It is also hypothesized that the high frequency will reach SNR of equal or greater magnitude than the other frequencies, because it works in a range that has less interference from spontaneous brain activity. With reference to attention, we propose that the attentional capacity of the subjects will be related to the amplitude of the SSVEPs obtained and, therefore, to their SNR.

The practical orientation of the work lays in the possibility to contribute to the construction of simple BCI systems that are easy to apply and quick to learn. It is possible to advance in this last point, by using the SSVEP paradigm, which is based on a purely sensory process, which needs no more condition than that of paying attention to the stimuli. In terms of easy application, we intend to be able to use a configuration of only two electrodes, which is why we will analyze which generate the most SNR out of the six we will measure.

## 2. Materials and Methods

### 2.1. Data Acquisition

After preprocessing and debugging the data, 42 university students of whom 31 (73.8%) were female and 11 (26.2%) were male, participated in the experiment in exchange for academic compensation. The average age was 19.12 years (sd = 1.64). All participants had normal vision. Color blindness, a history of past or present epileptic seizures, and/or severe migraines had been considered as exclusion criteria. All participants had been informed and had read and signed a consent that guarantees the confidentiality of the data and the freedom to participate as well as to leave the test at any time and without penalty. Signed consents are kept in a specific file. All procedures performed involving human participants were in accordance with the Ethical Commission for Experimental Research of the Universitat de València (https://www.uv.es/ethical-commission-experimental-research/en/commission/comission.html), that considers the Spanish and European legislations, the Ethical Declarations of Helsinki and Tokyo, the Bioethical Declaration of Gijón (24 June 2000) and the recommendations of the World Health Organisation.

A flexible EEG headcap (Enobio 8, Neuroelectrics company, Barcelona, Spain) was used to register EEG signals. It includes 8 easy-to-apply dry electrodes (no electroconductive gel required), and wirelessly communicates with a computer in real time, via Bluetooth.

**Figure 1:**
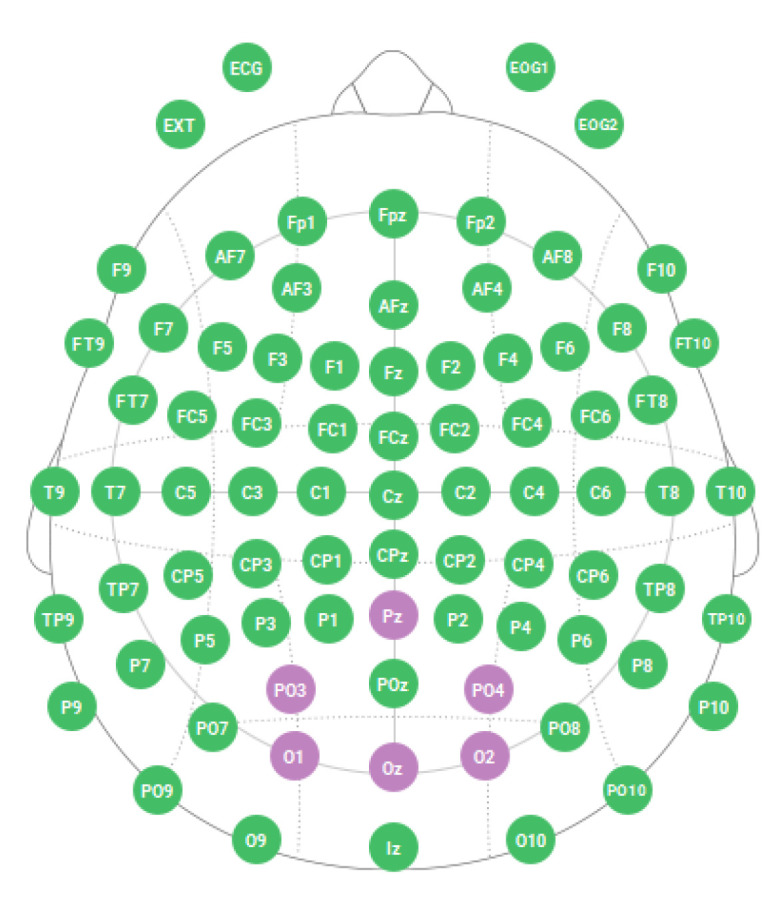
Disposition of the 6 electrodes used according to the international 10–20 system for electrode placement.

One of the aims of the experiment consisted in finding the electrodes that generate a higher SNR, in order to be able to build simple BCI systems that are easy to apply and with as few electrodes as possible. To check this point, EEG activity was recorded at six electrodes, three occipitals: O1, O2, and Oz, and three parieto-occipital: PO3, PO4, and Pz (Figure 1), which are the points most frequently reported in the BCI literature with the SSVEP paradigm [21,26,38,39], to later select the two single ones that generate the most activity. If we select only one, and it fails, it compromises the operation of the entire system, so we aimed to select and work with the average of two.

### 2.2. Visual Stimulation

**Figure 2:**
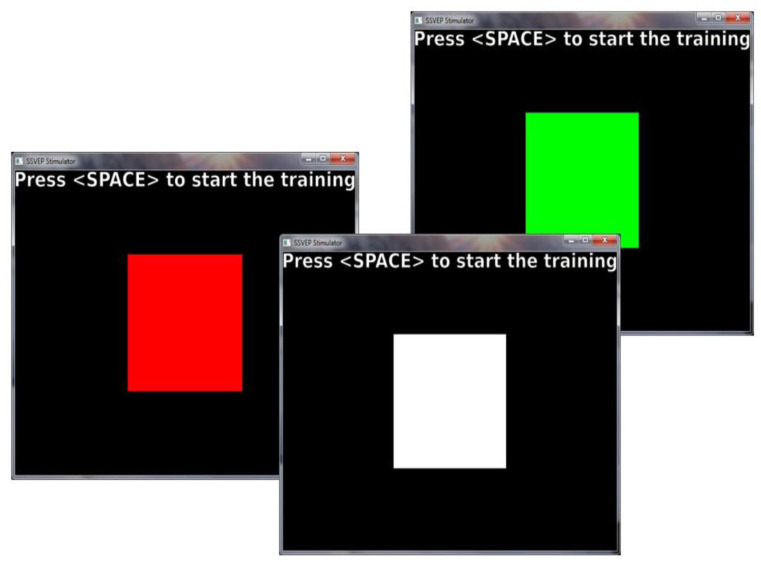
Square stimuli with the three colors presented during the experiment.

The Openvibe free software version 1.3.0 (Institut National de Recherche en Informatique et en Automatique, France) has been used for stimuli presentation and signal recording. This software allows to manage BCI experiments and has everything which is needed to communicate with the Enobio 8G helmet. It runs on a laptop with an Intel Core I7 processor and Windows 10 operating system, which has an auxiliary display with a refresh rate of 60 Hz. This frequency is necessary, because it must be a multiple of the flashing frequencies of the stimuli presented. The computer runs the program, presents the stimuli on the auxiliary screen—which only the participant can see—and receives and records the data sent by Enobio, via Bluetooth. Visual stimuli consist of square figures presented on an auxiliary screen with a refresh rate of 60 Hz. They can come in three colors: red, green, and white (Figure 2), and flash at three different frequencies: 5, 12, and 30 Hz. (low, medium and high, respectively) [40], which gives rise to nine experimental conditions resulting from crossing the three levels of the two variables (Table 1). We will name the colors red, green, and white as C1, C2, and C3 respectively, and frequencies 5, 12, and 30 as F1, F2, and F3.

### 2.3. Experimental Protocol

Participants were received and informed about the test, before reading and signing the informed consent. The preparation of the participant consisted of placing the Enobio helmet and performing an adjustment test of the electrodes. They then answered some questions about demographic data and psychoactive substance use. This preparatory part took about 15 min, and they were ready to start the experiment. The experiment took place in a quiet, low-light cabin, where participants sat comfortably at a distance of 60 cm of the auxiliary screen on which the stimuli were presented. All participants went through all the experimental conditions three times during the same session, so this is an intra-subject study. The order of presentation of the stimuli was completely random, both for the three series presented to each participant and for the series between participants. Each stimulus turned on and off intermittently for 10 s, and the time lapse between stimuli was 30 s. Participants were instructed to pay close attention to the square while blinking, and to avoid moving and blinking for 10 s. For the remaining 20 s between the end of one flash of the stimulus and the beginning of the next, they could blink and discharge any eye or body tension. At the end of each series, the participants stood up and were free to modify their position for three minutes. This part of the experiment took about 25 min.

To assess the attentional capacity of the participants, a standardized test was used: the Conner’s Continuous Performance Task version 2 (CPT-II) [41]. It is a behavioral task that measures reaction times, omission errors, and commission errors by responding with a click to the presentation of letters on a screen. Specifically, all letters are answered with the press of the space bar, except the letter X, which must not be answered. It is therefore a computerized test that has been run on a laptop where it was installed.

After the presentation of the three series of stimuli, the participants took the standardized test of attention with the CPT-II test that was installed on a different computer. Letters appeared sequentially on the computer screen, and participants were instructed to answer all letters except the X with a press of the space bar, while they do not have to answer when it comes to the X. The test is divided into six series of letters, and the time of appearance of the letters varied throughout each series. At the end of this test, which lasted about 14 min, the experiment concluded.

The whole session, including information and preparation of the subject, the presentation of the three sets of stimuli, and the completion of the attention test, lasted approximately one hour (Table 2). The sessions were held with a maximum of three per day, in a reduced afternoon time slot from 15:30 to 18:30 (first session from 15:30 to 16:30, second session from 16:30 to 17:30, and third session from 17:30 to 18:30) to avoid a possible effect of the participants’ circadian rhythms on the results of the experiment.

### 2.4. Electroencephalographic (EEG) Signal Processing

Once the tests were finished, the processing of the recorded signals was done with the proprietary software MATLAB version 2017b (https://www.mathworks.com), through the use of code sequences specifically developed to preprocess the signals, and then, to obtain all the SNR of interest, for all electrodes. The recorded signals went through two filters, a 3 to 80 Hz bandpass, and a 50 Hz slot filter for line noise. The data were visually inspected for erroneous or artifact records that might affect the quality of the SNR results to be calculated. Subsequently, the vectors (numerical lists) containing the data for each subject, condition, and electrode were truncated to extract 8 s records corresponding to the blinking at each stimulus. The first and last of the 10 seconds of presentation were discarded. These 8 s were segmented into 7 two-second overlapping windows (each one with the next one) 50% of the time. To each window, the fast Fourier transform (FFT) was applied, and the results were averaged, according to the Welch’s method. Welch’s method combines windowing and averaging for calculation of the power spectral density (PSD) of the signal, resulting in a smoother spectrum. The data resulting from this process were normalized considering the total spectral power equal to 1. Since the sampling frequency was 500 Hz, and the window had a length of 1000 samples (2s), the resolution of the FFT was 0.5 Hz. To obtain the SNRs, the first and second harmonics of each frequency were considered: 5 and 10 Hz for low frequency, 12 and 24 Hz for the average, and 30 and 60 Hz for high frequency. To calculate the SNR the following equation was used, where y(f) is the magnitude of the signal at frequency f, *n* is the total number of samples of the signal window and k was set to 4 (i.e., two frequencies on each side) [40,42].

$$SNR\;=\;\frac{n\;*\;y\left(f\right)}{\sum_{k=1}^{n/2}\left[y\left(f+0.25\;*\;k\right)+y\left(f-0.25\;*\;k\right)\right]}$$

This equation reveals the relation between the amplitude of the harmonic of interest and the average of the k neighboring frequencies.

**Figure 3:**
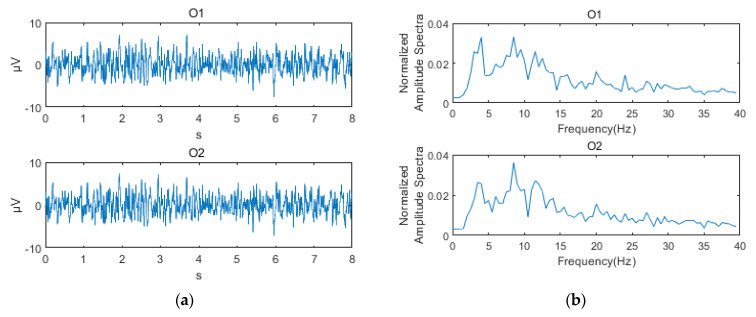

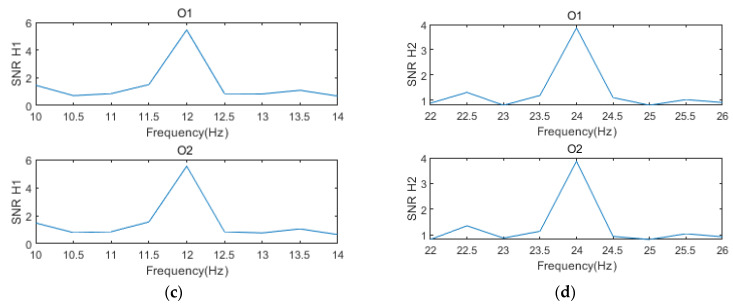
Stages in the processing of the temporal series to obtain the signal-to-noise ratio (SNR): (**a**) electroencephalographic (EEG) time course; (**b**) power spectral density; (**c**) harmonic 1 SNR; (**d**) harmonic 2 SNR.

Finally, the resulting SNRs were passed onto a logarithmic scale in decibels (dB). Since each participant went through all the experimental conditions three times in a random order, of the three SNRs corresponding to each condition, the average is calculated in one SNR per condition and participant. Prior to the condition analysis, an activity analysis of the electrodes was performed, which showed a higher activity at the occipital electrodes. Thus, for subsequent analyzes, the SNRs corresponding to the O1 and O2 electrodes were averaged, as established by one of the objectives of the work (Figure 3).

### 2.5. Statistical Evaluation

Among the available statistical analysis, analysis of variance (ANOVA) is a well-known and widely used procedure. In a data set, it aims to estimate the proportion of variance in the set due to one or more variables of interest, and the one due to random differences in subjects or groups. It can be performed for independent measures (different groups) or related measures (same groups in different time or condition). The result of this proportion is given as an index F and, moreover, ANOVA provides a statistical significance for this index. Therefore, due to its very nature and accepted validity for data analysis, ANOVA has been chosen for the primary evaluation method of our data.

With the SNRs for all participants and conditions, after averaging the results of the three presentations per participant, and the activity of the O1 and O2 electrodes, bifactorial normality and repeated measures ANOVA tests were performed in order to estimate the effect of the color and frequency variables on the resulting SNRs. Correlation analyses have also been performed between SNR measures by participant and attention measures obtained with the CPT-II test, consisting of four of the variables provided: reaction time, omission errors, commission errors, and general confidence index. Statistical variance and correlation tests have been performed on the SNR data obtained, with data about attention, by means of proprietary SPSS software (IBM), version 22.

## 3. Results

**Figure 4:**
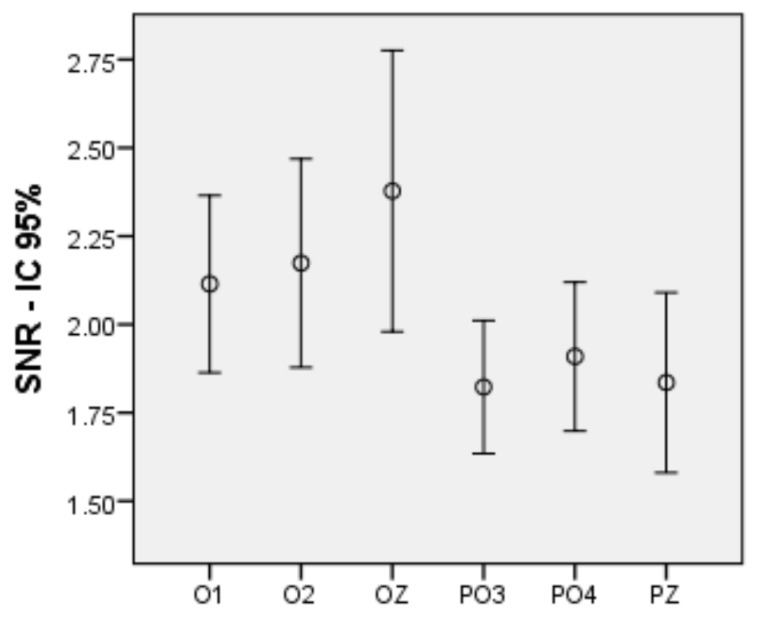
SNR means for the 6 electrodes measured with 95% confidence intervals.

Firstly, an intra-subject bifactorial ANOVA was performed in order to identify which electrodes were generating a higher SNR. This first step is decisive insofar as, for the subsequent analyses, we will rely on the two electrodes that generate the higher activity. The two factors are Lobe, with two levels: occipital and parieto-occipital, and Hemisphere, with three levels: left, right and medial. The ANOVA results indicate an effect of the Lobe factor (*F* (1, 41) = 17.990, *p* < 0.001, η^2^ = 0.305) and no effect of the Hemisphere factor. There is an effect of Lobe, but not of Hemisphere. Thus, the electrodes of the occipital lobe generated signals with more SNR than those of the parieto-occipital lobe. So, it seems preferable to work with the occipital ones. Between O1, O2 and Oz there is no statistical difference. Thus, if we aimed to use only one electrode, we would choose Oz, but as we aimed to use two for improved performance, we will rely on the average of the electrodes O1 and O2 to obtain the SNRs that will be used in all the analyses that follow. Figure 4 shows a graph with the SNR averages for the 6 electrodes.

For the analysis corresponding to the SNRs of the first harmonic, we subjected the 9 experimental conditions to normality tests. All conditions adjust to normal, except C3F1 (low-frequency white) and C3F3 (high-frequency white).

**Figure 5:**
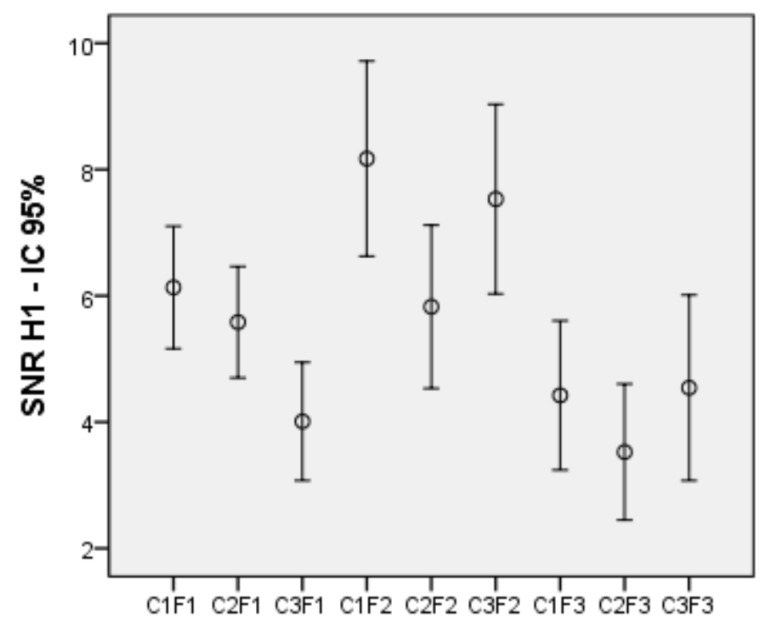
SNR means for the 9 conditions of the 1st harmonic with 95% confidence intervals.

To test the effect produced by the variables color and frequency on the SNR of the first harmonic, an intra-subject bifactorial ANOVA on the 9 conditions was performed. Three levels: red, green and white, were considered for the color factor, and three: 5, 12 and 30 Hz, for the frequency factor. The results show an effect of both the color (*F* (2, 82) = 11.718, *p* < 0.001, η^2^ = 0.222) and the frequency (*F* (2, 82) = 15.363, *p* < 0.001, η^2^ = 0.273), as well as an interaction between both of them (*F* (4, 164) = 7.266, *p* < 0.001, η^2^ = 0.151). Figure 5 shows a graph with the SNR averages for the 9 experimental conditions of the first harmonic.

Since there is an interaction between factors, the simple effects have been analysed as it is shown in Table 3 (color) and Table 4 (frequency).

Regarding the simple effects of color, at 5 Hz, red and green generated more SNR than white (*p* < 0.001 for both), and there were no differences between the first two. At 12 Hz, red generated more SNR than green (*p* < 0.001) and white more than green (*p* = 0.006); No differences between red and white were found. At 30 Hz, no differences between the three colors appeared. Regarding the simple effects of frequency, with red, the average frequency generated more SNR than the low (*p* = 0.004) and the high one (*p* < 0.001). The low frequency also generated more SNR than the high one (*p* = 0.027). For green, both the low frequency (*p* = 0.008) and the medium frequency (*p* < 0.002) generated more SNR than the high one, with no differences between the first two. Finally, for white, the average frequency generated more SNR than the low and high ones (*p* < 0.001 for both). In addition, the effect of the color and frequency variables on the SNR of the second harmonic signals was analysed. First, the 9 conditions corresponding to the second harmonic were subjected to normality tests. All conditions were normal, except C1F3 (high frequency red).

**Figure 6:**
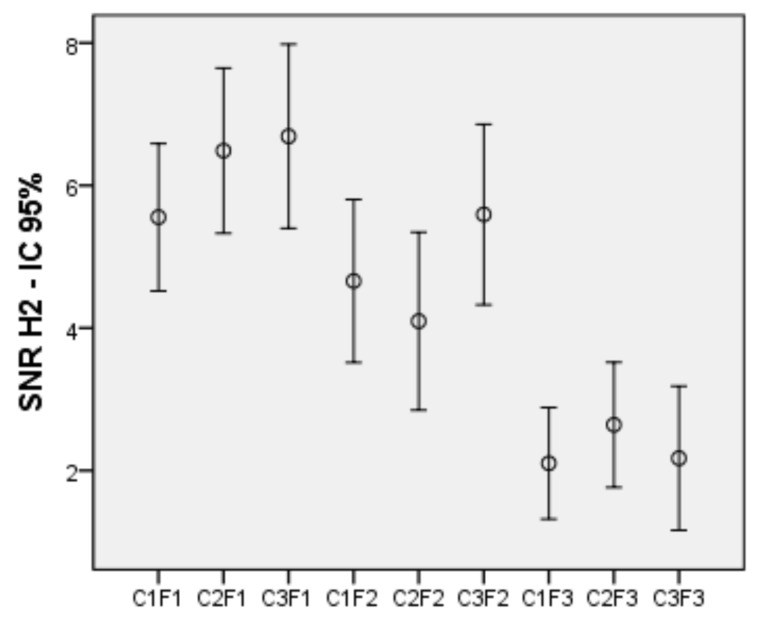
SNR means of the 9 conditions for the 2nd harmonic with 95% confidence intervals.

Again, a bifactorial intra-subject ANOVA was applied to color and frequency factors, which showed a significant effect of color (*F*(2, 82) = 6.033, *p* = 0.004, η^2^ = 0.128) and of frequency (*F*(2, 82) = 39.232, *p* < 0.001, η^2^ = 0.489), as well as an interaction between both factors (*F*(4, 164) = 4.107, *p* = 0.003, η^2^ = 0.091). Figure 6 shows SNR means for 9 conditions of the 2nd harmonic.

Since, again, there was an interaction between factors, the simple effects were analysed as shown in Table 5 (color) and Table 6 (frequency).

**Figure 7:**
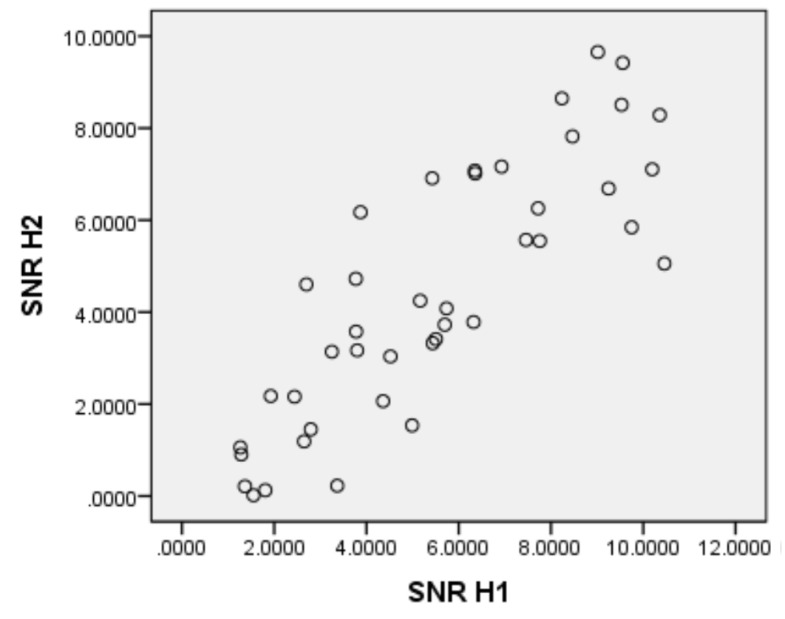
Scatter plot for SNR of the 1st and 2nd harmonics.

When considering the simple effects of the color variable, at 10 Hz, both white (*p* < 0.001) and green (*p* = 0.045) generated more SNR than red, with no differences between the first two. At 24 Hz, white generated more SNR than green (*p* < 0.001) and no differences between red and white or red and green were found. And at 30 Hz there are no differences between the three colors. As for the simple effects of the frequency variable, with red, both 10 Hz (*p* < 0.001) and 24 Hz (*p* < 0.001) generated more SNR than 60 Hz, with no differences between the first two. With green, 10 Hz generated more SNR than 24 Hz (*p* = 0.001) and 60 Hz (*p* < 0.001), while 24 Hz also generated more SNR than 60 Hz (*p* = 0.024). Finally, with white, both 10 Hz (*p* < 0.001) and 24 Hz (*p* < 0.001) generated more SNR than 60 Hz, with no differences between the first two. The visual inspection carried out while preprocessing the data showed that the first harmonic was not always accompanied by the following ones. Some subjects presented the first harmonic in absence of the second, while others present the second in absence or with very little amplitude of the first. For this reason, it is interesting to know the relationship between the activity of the first and second harmonics, and thus, a correlation analysis between the SNR corresponding to the first harmonic and that of the second was performed. The two distributions were adjusted to normality and a high correlation (*r* = 0.839, *p* < 0.001) was obtained between the two harmonics (Figure 7 shows the scatter plot).

To verify the existence of relationships between the SNR of the signals measured in the SSVEP task and the attentional capacity of the participants, correlation analyzes were performed between the SNR of the harmonics in the 9 experimental conditions and 4 variables provided by CPT-II. These are the reaction time, omission errors and commission errors, all three considered to be valid measures of sustained attention capacity, as well as the general confidence index, a global probabilistic measure indicative of the clinical/non-clinical status of the participant, in terms of attention and impulsivity disorders. Analyses showed a negative correlation between SNR of the 1st harmonic signal and reaction time, for all three colors and only for low frequency (Table 7 indicates the results of correlation analyses). No correlations were found for the other frequencies and attentional variables.

Regarding the results of the questions contained in the participant questionnaire, 100% of the subjects did not consume alcohol during the day of the test. 42.9% drank coffee, while 57.1% did not. 7.1% had slept 5 h or less, 64.3% had slept 6 or 7 h, and 28.6% had slept 8 h or more. Finally, 38.1% took the test from 15:30 to 16:30, 40.5% from 16:30 to 17:30, and 21.4% from 17:30 to 18:30.

## 4. Discussion

Several questions related to the possibility of building simple BCI systems that can be easy to apply and would need little training were addressed in this study. Regarding training, the SSVEP paradigm has been chosen, as it rests on a purely sensory neural process that only requires visual attention in order to work, thus being the paradigm that needs the least training. For people with disabilities, a multi-electrode setup that uses electroconductive gel can be very uncomfortable to the point of being impossible. A setup with a couple of dry electrodes that can be placed on the head—even with the aid of some kind of tape—may be very promising. We wondered which would be the best places and electrodes to get a good detection and discrimination of the measured frequencies. Our results show that the electrodes in the occipital cortex can generate the highest SNR and all subsequent analyses were based on measurements obtained at the O1 and O2 electrodes.

Next, we focused on the effect of color and frequency on the SNRs obtained. We wanted to know if white would produce results similar to the ones obtained with red, which would make it possible to dispense with the risks associated with the latter color. Also, we were willing to know if high frequencies would produce results equal to or higher than the low and medium ones, since that outcome would allow us to avoid working in an area of the spectrum with greater spontaneous brain activity. The ANOVA results for the two color and frequency factors show a significant effect of the two variables, both for the first harmonic of the presented frequencies and for the second, as well as an interaction between the two factors. We analyzed the simple effects and found significantly different effects for the two harmonics.

### 4.1. First Harmonic–Color

Focusing on the first harmonic, it can be observed that there are no differences between red and green for the 5 Hz frequency, while both do produce greater SNRs than white. In contrast, at a frequency of 12 Hz there are no differences between red and white, and both produce higher SNRs than green. At a frequency of 30 Hz, no color differences were found. Authors in [21,29] found that the red color is the one that produces the best results. Our results, however, suggest that working with a medium frequency—according to the classification of [19]—white can be used instead of red, while equivalent results can be obtained in discrimination capacity, on the basis of frequency analysis and SNR. In contrast, with a low frequency of 5 Hz, the SNR for white decreases significantly, and better results can be reached with both red and green. One possible reason for this drop would be that the target would be especially difficult to meet at a low frequency. Low and medium frequencies are known to cause more visual fatigue [19,28], and white could intensify the situation in the low-frequency condition.

However, in both cases we could do without the red color and use green at low frequencies as well as white at medium frequencies. For high frequency, there is no difference between the colors, so both, white and green can be used instead of red. The results obtained partially support our hypothesis that white would generate results equivalent to red, and allow us to dispense with it, at least for the medium and high frequencies.

### 4.2. First Harmonic—Frequency

Regarding the effect of the frequency, our results indicate that the medium frequency produces higher SNR than the low and high ones, for red and for white, as well as higher SNR than the high for green. The high frequency produces the lowest SNR of the three for red and green colors, while no differences were found with the low frequency for white. With these results, the medium frequency is more favorable, insofar as it produces higher SNR than low and high in all cases, except with the green color, which is equal to the low. This result contradicts our hypothesis that high frequency might be more advantageous. The results mentioned in the review by [19] suggested that the amplitude of SSVEPs would be greater at medium frequencies, and would decline, to a greater or lesser extent depending on the color, at low and high frequencies. Our results are along the same lines. The aforementioned review proposed, however, that high frequencies should be further investigated, because they could present advantages due to decreasing interference with spontaneous brain activity, rather than the low and medium ones, although the amplitude of SSVEPs is reduced at these frequencies. Our results put the high 30 Hz-frequency at a disadvantage. Only for white does it look like it can produce an SNR equivalent to that of the low frequency. This might be explained by the fact that, as has been shown, the white color at low frequency is at a disadvantage compared to the other two colors. The explanation for these high-frequency SNR declines could be that, although the noise activity is lower, the signal level decreases with frequency, as noted in the review, and perhaps does so in a greater proportion than noise, thereby eventually producing a smaller SNR amplitude. Therefore, with respect to the frequency of the first harmonic, the results of the revision are confirmed, and we find no confirmation for the hypothesis that high frequencies could be advantageous because of their ability to generate a higher SNR than lower ones.

### 4.3. Second Harmonic—Frequency and Color

Focusing on the second harmonic, we found that the low frequency produces SNRs higher than the medium one, with the green (the same for red and white), while the medium frequency produces higher SNRs than the high one for all colors. Regarding color, white and green produce higher SNR than red at low frequency, while white along with red produce higher SNR than green at medium frequency. There are no color differences at high frequency. In short, the amplitude of the SNR falls with the frequency, a fact that we have already been able to check with the analysis of the first harmonic, and that now can be seen in more depth because, for the second harmonic, we started from an average frequency (10 Hz), for the first level of the variable, and we moved to high frequencies, both for the second and the third levels of the variable (24 and 60 Hz, respectively). The amplitude of the SNR also has an advantage for white at low and medium frequencies. In short, we have found advantages in the second harmonic with the use of white, and we got the highest SNRs, with the low frequency. It should also be noted that for this second harmonic, the effect of frequency (η^2^ = 0.489) is much greater than the effect of color (η^2^ = 0.128).

### 4.4. Correlation between Harmonics

In order to design BCI devices, some studies choose to discriminate only the first harmonic in classification tasks [43], while others discriminate both the first and the second ones [44], or even three [39]. Using two harmonics for classification could present some advantages for decision making. Classification tasks must take decisions about the presence and identity of harmonics, in order to act on the control system of physical devices. Thresholds should be set in the SNR to discriminate harmonics, and limitations on the reliability and robustness of this process can lead to the emergence of false positives. With our data, we can observe a high correlation between the SNRs produced by the two harmonics, which indicates that, in general, the presence of one harmonic is accompanied by the other, although visual inspection shows that not all participants present the second harmonic along with the first, and there are even times when some participants present only the second harmonic and not the first one. An advantage of using two harmonics would, therefore, be to have more information available for decision-making purposes in classification tasks. The SNR measurement of the first harmonic could be reinforced with the measurement of the second, thus facilitating decision making.

### 4.5. Correlation with Attention

Regarding the relationship between attentional capacity and amplitude of the produced SSVEPs, correlation analyses were performed between the variables corresponding to the SNR of the 9 experimental conditions and the variables reaction time, omission errors, commission errors, and confidence index, all of them provided by the CPT-II test. Moderate correlations were found between Reaction Time and SNR amplitude for all three colors, at low frequency, while no correlations were found for the remaining conditions and variables. The negative sign of the correlations indicates that higher SNRs are related to lower reaction times, an observation that is consistent with the effect of attention on both tests. A higher sustained attention capacity would be reflected in lower reaction times in the CPT-II task, as well as a greater amplitude of the evoked potentials in the SSVEP task which would, therefore, provide more SNR. It is interesting that this correlation appears only with low frequency. One possible explanation is that the observation of stimuli that flicker at low frequency could require more attentional effort and concentration, due to the fatigue and discomfort caused by them, when compared to stimuli of higher frequencies, as it has already been mentioned. Participants with a higher attentional ability might be better prepared to make this effort while maintaining concentration, and would perform better on the SSVEP task than subjects with less ability. Finally, the information collected on sleeping hours or consumption of psychoactive substances has not been shown to have any relation to the SNR obtained.

### 4.6. Studies Comparison and Limitations

Out of the 12 reviewed references, this study is the only one that worked with the Enobio interface; 42 subjects were analyzed, while in the other references subjects were between 1 and 20. Number of electrodes used is two in [38] and [21]. This study analyzed the signal at the O1 and O2 electrodes. See Table 8 for a summary.

Out of 12 references reviewed, 4 use square stimuli, 4 checkerboard and the others have different stimulus type. The low and medium frequency bands are used in 11 references and 3 also use high frequency. Monitors, light-emitting diodes (LEDs) and tablets are used in the screen type. With the square stimulus, the screen used is a monitor. Comparing the present study with references [21,26] that have the same screen (liquid crystal display (LCD) monitor) and stimulus type (square), it can be concluded that in the present study a better response was found in the frequency of 5 Hz for the red and green colour, at 12 Hz for the red and white colour, and at 30 Hz the three colours had the same response. In [26] there is a better response of white at low and medium frequencies, followed by the colours gray, red, green and blue. In [21] the study was done at a low frequency with a good impact on colour red and orange. See Table 9 for a summary.

The main limitations of the present work have to do with the use of dry electrodes and with the age range of the sample. With regard to the first issue, the use of wet electrodes is likely to improve the signals obtained and the corresponding SNR. However, it should be noted that, on the one hand, we aim to build BCI systems that are easy to apply, with few electrodes and dry, so that resourcing to use wet electrodes would take us away from our target. On the other hand, the effect of using dry or wet electrodes would affect all conditions and subjects equally. It is not foreseeable, therefore, that differences in activity analyses and color and frequency effects could occur. Regarding the age, the range in our sample is quite short and, consequently, we cannot know what effect it might have on the variable of interest in this study. Other studies should be designed with subjects of different ages, which opened up the possibility of clarifying this issue. Also, future research should consider the option of working with multiple mid-band frequencies at the same time, because, as has been noted, the results of this and other studies suggest that they might produce the best results. However, it would be necessary to study how many medium frequencies can be used at the same time, bearing in mind that the proximity of harmonics in a limited frequency band could make discrimination difficult. Also, in case the use of low frequencies was forced, it could be considered whether some form of mindfulness training, such as mindfulness or neurofeedback, could be able to improve the performance obtained.

Finally, a matter of paramount importance is to continue at the point where it ends here: to build complete systems with order classification tasks and control of devices, and to implement the conclusions reached in order to check how they work. In this context, high frequencies could continue to be studied; while there is evidence that they produce less SNR, perhaps working in a frequency band in which spontaneous brain activity is lower would help reduce the number of false positives. All of these issues could be verified in the practice of complete systems with the measurement of performance, or through the percentage of successes for each participant in a task.

## 5. Conclusions

At this point, the conclusions that can be draw from the results of this work are:

1. The average frequency produces the best SNRs, followed by the low, and finally the high frequencies.
2. Both white and red can be used at a medium frequency, and both green and red at a low frequency, while at high frequency there are no differences between the colors.
3. Therefore, it is possible to dispense with the use of red in order to avoid possible associated risks.
4. Detection and discrimination of two harmonics could provide advantages in classification tasks, because they support each other, and if it is necessary to work with low frequencies, attentional ability may be relevant for good results.

The BCI area is relatively new with a still very long way to go. Applying it to improving the quality of life of people with disabilities makes it a field of great humanitarian interest and potential. That said, for BCI systems to achieve the purpose for which they are intended, mechanisms are needed to read the intentions of the participants to act on the various devices with maximum reliability and robustness. Basic studies such as the one presented here are necessary to correctly understand how to extract useful information from the neural electrical activity of the participants, so that they are able to interact directly with the outside world. All this is with the aim of creating solid foundations on which we can contribute to the improvement of the evolution of this fascinating field of basic and applied study.

## Figures and Tables

**Table 1:** Experimental conditions resulting from combining stimulus color and frequency.

Colour/Frequency	5 Hz	12 Hz	30 Hz
Red	C1F1	C1F2	C1F3
Green	C2F1	C2F2	C2F3
White	C3F1	C3F2	C3F3

**Table 2:** Duration of the different parts of the experiment.

Experiment Stage	Time (min)	Activities
Subject preparation	15	- Filling out consent form - General information (posture, stop the experiment) - Initial questionnaire - Electrode placement
Experiment session	25	- SSVEP stimuli
CPT-II Test	15	- Standardized test of attention
Total experiment time	55	

**Table 3:** Simple effects of the color variable on SNR for the 1st harmonic, for the three frequencies.

Frequency	Colour Analysis
5 Hz	*F*_(2, 82)_ = 15.912, *p* < 0.001, η^2^ = 0.28
12 Hz	*F*_(2, 82)_ = 12.17, *p* < 0.001, η^2^ = 0.229
30 Hz	Not significant

**Table 4:** Simple effects of the Frequency variable on SNR for the 1st harmonic, for the three colors.

Colour	Frequency Analysis
Red	*F*_(2, 82)_ = 17.86, *p* < 0.001, η^2^ = 0.303
Green	*F*_(2, 82)_ = 7.973, *p* = 0.001, η^2^ = 0.163
White	*F*_(2, 82)_ = 12.177, *p* < 0.001, η^2^ = 0.229

**Table 5:** Simple effects of color on SNR for the 1st harmonic for the 3 frequencies.

Frequency	Colour Analysis
10 Hz	*F*_(2, 82)_ = 5.229, *p* = 0.007, η^2^ = 0.113
24 Hz	*F*_(2, 82)_ = 6.514, *p* = 0.002, η^2^ = 0.137
60 Hz	Not significant

**Table 6:** Simple effects of the variable Frequency on the SNR of the 1st harmonic in the 3 colors of the experiment.

Colour	Frequency Analysis
Red	*F*_(2, 82)_ = 22.884, *p* < 0.001, η^2^ = 0.358
Green	*F*_(2, 82)_ = 23.497, *p* < 0.001, η^2^ = 0.364
White	*F*_(2, 82)_ = 35.086, *p* < 0.001, η^2^ = 0.461

**Table 7:** SNR correlations for the three colors at low frequency and reaction time (RT) at CPT-II task.

Colour	Correlations
Low frequency red SNR and RT	*r* = −0.453, *p* = 0.003
Low frequency green SNR and RT	*r* = −0.393, *p* = 0.01
Low frequency white SNR and RT	*r* = −0.359, *p* = 0.02

**Table 8:** Characteristics of adquisition device and electrodes.

Study	Interface	Electrodes	Electrodes Placement	Subjects
Present study	Enobio	8/2	Cz, O1, O2, PO3, Oz, PO4, Pz	42
Cao et al. 2012 [26]	g.USBamp, Guger Technologies	6	POZ, P3, P4, OZ, O1, O2	5
Chu et al. 2017 [21]	Biosemi	16/2	POZ y OZ	15
Chen et al. 2019 [45]	Neuroscan amplifier	64/9	Pz, POz, Oz, PO3, PO4, PO5, PO6, O1, O2	12
Floriano et al. 2018 [40]	Grass 15LT Amplifier and NI-DAQ Pad6015	3	OZ, TP9, TP10, A2	12
Yan et al. 2017 [27]	g.USBAmp, Guger Technologies	15	O1, Oz, O2, PO7, PO3, POz, PO4, Cz, P1, Pz, P2, CP3, CPz, CP4 y Fz	9
Szalowski and Picovici. 2016 [46]	Emotiv Epoc Headset	14	AF3, F3, F4, AF4, FC5, FC6, F7, F8, T7, T8, P7, P8, O1, O2	1
Szalowski and Picovici. 2019 [47]	Emotiv Epoc Headset	14	AF3, F7, F3, FC5, T7, P7, O1, O2, P8, T8, FC6, F4, F8, AF4	1
Chien et al. 2017 [48]	64-channel Quik-Cap. EEG amplifier (SynAmps2 model 8050, Neuroscan)	64/9	P1, PZ, PO3, POZ, PO4, O1, OZ, O2	10
Tello et al. 2015 [29]	BrainNet-36 equipment	12	P7, PO7, PO5, PO3, POz, PO4, PO6, PO8, P8, O1, O2, Oz	20
Evain et al. 2016 [49]	g.USBAmp, Guger Technologies	6	CPz, POz, Oz, Iz, O1, O2	12
Cheng et al. 2001 [38]	Two monopolar channels	2	O1, O2	-

**Table 9:** Characteristics of stimuli.

Study	Frequencies (Hz)	Screen	Shape	Stimuli Number	Colour Stimuli	Colour Evaluation
Present study	5,12,30	LCD monitor	Square	3	Red (R) Green (G) White (W)	5: R = G > W 12: R = W > G 30: R = G = W
Cao et al. 2012 [26]	17.14, 15, 13.33, 12, 10.9, 10, 9.23, 8.57, 8, 7.5	LCD monitor	Square	Offline:5/Online:16	Gray, Red, Green, Blue, White	High performance is white, followed by gray, red, green and blue stimuli.
Chu et al. 2017 [21]	10	Monitor	Square	10	Light purple, Dark purple, blue, Light green Dark green, Yellow, Orange Red, Brown, White	Violet colour had the least influence. Red and orange colour has stronger impact.
Chen et al. 2019 [45]	Low: 6, 8, 10, 12, 14, 16, 18, 20, 22 High: 24, 26, 28, 30, 32, 34, 36, 38, 40	LCD Monitor	Square	1	White	Stimulus waveform (sine or square)
Zhu et al. 2010 [19]	4–50 Low: 1–12 Medium: 12–30 High: 30–60	LCD Monitor	Square	-	White, Black	-
Floriano et al. 2018 [40]	5, 10, 15, 20, 25, 30, …, 65	Light emitting diodes (LED)	Checkboard	4	Red-Green (R-G) Green-Blue (G-B) White	15–25: GR > W 30–40: GB > W 55–65: W > RG = RB
Yan et al. 2017 [27]	<15	Monitor	Checkboard	2	Red-Green White-Black	Red-green in low frequency (<15 Hz) produced higher power and recognition accuracy than black-white.
Szalowski and Picovici. 2016 [46]	10	LCD monitor	Checkboard	11	Blue-White, Blue-Magenta, Blue-Red, Green-White, Green-Blue, Magenta-White, Green-Magenta, Red-White, Red-Green, Red-Magenta.	Cleanest 10 Hz peak: Red-White. Then Black-White, Blue-White, Green-Blue, Magenta-White. Poor response of blue-red
Szalowski and Picovici. 2019 [47]	10	Tablet 12.9”	Checkboard	33	RGB (Red-Green-Blue). 33 combinations: first color (green, red, blue, yellow, cyan and magenta with white, black), second color (gray)	Significant signal gain from the use of colour flickers compared to greyscale flickers.
Chien et al. 2017 [48]	32 40	Light emitting diodes (LED)	Projected onto a viewing screen	2	Red-Green Red-Blue	Dual-colour lights flickering (R/G–R/B) achieved a greater detection accuracy with little or no flickering sensation.
Tello et al. 2015 [29]	8, 11, 13, 15	Light emitting diodes (LED)	Box	4	Red, Green, Blue, Yellow	Red is less comfortable. Order of choice was: green, blue and yellow
Evain et al. 2016 [49]	10, 12, 15	Monitor	Circle	3	Green, Orange, purple	No significant effect of colour on accuracy was found neither during training phase and end use.
Cheng et al. 2001 [38]	6.45, 7.23, 8.01, 13.87	LCD monitor	Block	1	Red, Green, Yellow	Stimulus method

## Data Availability

The data presented in this study are available on request from the corresponding author. The data are not publicly available due to privacy restrictions.
